# Outcomes of Primary Esophagectomy and Esophagectomy after Endoscopic Submucosal Dissection for Superficial Esophageal Squamous Cell Carcinoma: A Propensity-Score-Matched Analysis

**DOI:** 10.3390/cancers15235542

**Published:** 2023-11-23

**Authors:** Minjee Kim, Tae Jun Kim, Ga Hee Kim, Yeong Chan Lee, Hyuk Lee, Byung-Hoon Min, Jun Haeng Lee, Poong-Lyul Rhee, Jae J. Kim, Yang Won Min

**Affiliations:** 1 Department of Medicine, Samsung Medical Center, Sungkyunkwan University School of Medicine, Seoul 06351, Republic of Korea; minjee0803.kim@samsung.com (M.K.); tj23.kim@samsung.com (T.J.K.); lhyuk.lee@samsung.com (H.L.); jason.min@samsung.com (B.-H.M.); jh2145.lee@samsung.com (J.H.L.); pl.rhee@samsung.com (P.-L.R.); jaej.kim@samsung.com (J.J.K.); 2 Department of Medicine, Asan Medical Center, Seoul 05505, Republic of Korea; gahee.kim@amc.seoul.kr; 3 Biomedical Statistics Center, Research Institute for Future Medicine, Samsung Medical Center, Seoul 06351, Republic of Korea; conan_8th@naver.com

**Keywords:** esophageal cancer, endoscopic submucosal dissection, surgery

## Abstract

**Simple Summary:**

Currently, it is unknown whether secondary esophagectomy after endoscopic submucosal dissection (ESD) is comparable to primary esophagectomy. The aim of our retrospective study was to compare short- and long-term clinical outcomes between the two groups. Propensity matching was performed, and 34 patients in each group were compared. Comparing primary and secondary surgery, lymph node metastasis (LNM), overall survival (OS), disease-specific survival (DSS), and recurrence-free survival (RFS), were comparable between the two groups. Comparing the adverse events between the two groups, none of the patients in either group died within 60 days of treatment. There was no significant difference in the overall adverse events, but more early complications were observed in the primary surgery group than in the secondary surgery group. ESD and secondary esophagectomy can be recommended for patients with superficial esophageal cancer, as it does not compromise the outcomes of survival, recurrence, and complications.

**Abstract:**

Even though the conventional treatment for T1 esophageal cancer is surgery, ESD is becoming the primary treatment. Currently, it is unknown whether secondary esophagectomy after endoscopic submucosal dissection (ESD) is comparable to primary esophagectomy when considering outcomes in patients with T1 esophageal cancer. We compared short- and long-term clinical outcomes between the two groups. Primary surgery (esophagectomy) was performed in 191 patients between 2003 and 2014, and 62 patients underwent secondary surgery (esophagectomy) after ESD for T1 esophageal cancer between 2007 and 2019. Propensity matching was performed for age, sex, Charlson Comorbidity Index (CCI), location, pathology, degree of differentiation, tumor size, and invasion depth. Lymph node metastasis (LNM), overall survival (OS), disease-specific survival (DSS), recurrence-free survival (RFS), and post-operative complications were compared between groups. Sixty-eight patients were included after propensity score matching; LNM, OS, DSS, and RFS were comparable between the two groups. Comparing primary and secondary surgery, the respective LNM rates were 23.5% and 26.5%, 6-year OS 78.0% and 89.7%, *p* = 0.15; DSS were 80.4% and 96.8%, *p* = 0.057; and RFS were 80.8% and 89.7%, *p* = 0.069. Comparing the adverse events between the two groups, there was no significant difference in the overall adverse events. However, more early complications were observed in the primary surgery group than in the secondary surgery group (50% vs. 20.6%, *p* = 0.021). Secondary surgery did not increase the risk of LNM. The long-term outcomes were comparable. Therefore, attempts to perform upfront ESD for superficial esophageal squamous cell cancers are justified.

## 1. Introduction

Globally, esophageal cancer is the eighth most commonly diagnosed cancer and the sixth most common cancer leading to death [1]. Eastern and South Central Asia account for ~75% of new cases and deaths from esophageal cancer [2]. The conventional treatment for superficial esophageal cancer is surgery [3]. However, owing to the risk of complications of esophagectomy and low quality of life post-surgery [3], this procedure is not suitable for all patients [4,5].

Endoscopic submucosal dissection (ESD) is associated with a low risk of morbidity and mortality [6,7]. In Japan, ESD is the primary endoscopic treatment for superficial esophageal cancer, with overall better en bloc resection and local recurrence rates than endoscopic mucosal resection (EMR) [8]. ESD is gaining popularity in Korea, with thousands of procedures being performed yearly, as it allows en bloc resection of a lesion regardless of its size and location [9]. However, the procedure resects primary lesions and not the lymph nodes, necessitating the need for screening and monitoring lymph node metastasis (LNM) before and after the procedure [10,11,12]. Furthermore, even with a successful primary resection, surgical resection must be considered in case of recurrence and metastasis.

The increasing number of ESD procedures increases the number of cases of non-curative resection, which will inevitably increase the number of cases requiring secondary surgery. To proceed with secondary surgery, we need to establish whether this two-phase treatment will have any adverse effects on long-term outcomes. 

Thus far, no studies have compared the outcomes of esophagectomy and esophagectomy after ESD in terms of LNM, overall survival (OS), disease-specific survival (DSS), and recurrence-free survival (RFS). Here, we aimed to compare LNM and long-term outcomes of primary surgery versus ESD with secondary surgery for superficial esophageal cancer in a matched cohort.

## 2. Materials and Methods

### 2.1. Study Design 

This retrospective cohort study was conducted from January 2003 to December 2019 at the Samsung Medical Center, a tertiary institution in Seoul, Republic of Korea. We reviewed electronic medical records (EMRs) to obtain the variables for each patient. Patients who underwent either ESD followed by surgery or primary surgery for superficial esophageal squamous cell carcinoma (SESCC) at Samsung Medical Center were included. 

Esophageal ESD was performed when superficial esophageal cancer showed no evidence of submucosal (SM) invasion, distant metastasis, or LNM. Additional esophagectomy with ESD was indicated when there was incomplete resection or when the pathological reports were non-curative (presence of more than SM invasion and/or lymphovascular invasion (LVI)), even though complete resection was performed, or local recurrence within 6 months. Cancer patients with a risk of SM invasion, LNM, LVI, and circumference and size > 5 cm in diameter were treated with esophagectomy owing to the high risk of stricture after ESD. If the patient agreed to undergo ESD, ESD was performed even if there was a high risk of stricture. However, surgery was recommended if a refractory stricture (such as a circumferential lesion > 5 cm in length) was expected. 

**Figure 1:**
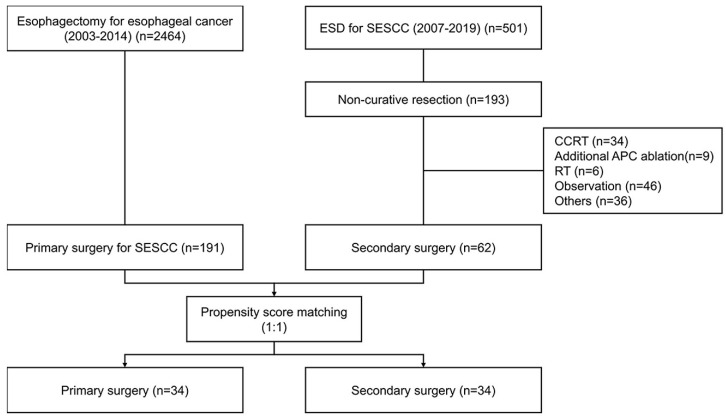
Patient flow diagram.

We enrolled 2464 patients who underwent esophagectomy between 2003 and 2014. In total, 191 patients underwent primary surgery with SESCC. Furthermore, 501 patients underwent ESD for SESCC between 2007 and 2019. Moreover, 62 patients underwent secondary surgery after non-curative resection of ESD. In this study, 1:1 propensity score matching was conducted 1:1, with 32 patients in each group (Figure 1). 

Clinicopathological factors, including age, sex, Charlson Comorbidity Index (CCI), tumor location, pathology, degree of differentiation, tumor size, invasion depth, LVI, and LNM, were assessed. The variables were based on the pathology of surgical resection in the primary surgery group and the combination of endoscopic resection and post-endoscopic surgical specimens in the secondary surgery group. Information, including surgery date, endoscopic resection date, recurrence date, recurrence type, death date, survival status, and cause of death, was obtained from hospital records and test results. The follow-up duration was defined as the duration between the date of the first procedure and the last survival date. For the ESD and surgery groups, the follow-up duration for survival was defined as the date from ESD to last survival. Duration of recurrence was defined as the time between surgery and the date of recurrence in both the primary and secondary surgery groups. 

This study was approved by the Institutional Review Board (IRB) of the SMC (IRB file number: SMC 2023-04-073-001). It was conducted under the Declaration of Helsinki. The board exempted informed consent because all data were analyzed anonymously. 

### 2.2. Diagnostic Workup and Procedure

For diagnosis, all patients underwent endoscopic evaluation before the intervention, including chromoendoscopy using Lugol’s dye spray method. Additionally, endoscopic ultrasound (EUS) was performed on most patients to confirm the diagnosis of superficial cancer. Furthermore, computed tomography (CT) was performed to identify distant organs or LNM. Surgery was recommended if SM invasion was suspected during the work-up (such as EUS or esophagogastroduodenoscopy) or if the stricture risk was high even though the invasion depth was restricted to the mucosa [13,14].

Esophageal ESD was performed using standard techniques. All procedures were performed under general anesthesia. After spraying with iodine solution, the cancerous lesion was identified, and each lesion was marked ~3 mm outside its margin with cautery using an electrosurgery unit. Circumferential mucosal precutting was performed using an SM injection. Various knives were used to dissect the SM layers. 

An Ivor Lewis or McKeown operation was performed for esophagectomy. Primary surgery was defined as curative esophagectomy performed as the first-line treatment for esophageal cancer, whereas secondary surgery was defined as esophagectomy after endoscopic resection. 

### 2.3. Outcomes

The primary outcome of this study was LNM, and the secondary outcomes were OS, DSS, RFS, and post-operative complications. LNM was defined as the presence of LNM in a surgical (esophagectomy) specimen. OS and DSS were determined based on survival from the date of intervention (surgery or ESD, whichever occurred first) until death. If the main cause of death was registered with the code for esophageal cancer in the Statistics Korea database, it was defined as death due to esophageal cancer. Recurrence-free survival (RFS) was defined as mortality and recurrence during the study period. Survival and recurrence data were collected until December 2022. Adverse events included early adverse events, such as pulmonary events, cardiovascular events, leaks/fistulas, wound problems (e.g., wound dehiscence and infections), vocal cord palsy, and other problems, and late adverse events, such as strictures. Early adverse events occurred within 30 days after surgery, and late adverse events occurred 30 days after surgery. Stricture was defined as the presence of dysphagia requiring intervention, such as the use of a bougie. 

### 2.4. Statistical Analysis

Continuous variables among baseline characteristics were analyzed using Student’s t-test, while categorical variables were analyzed using the chi-square test. OS, DSS, and RFS were analyzed using Kaplan–Meier curve analysis. Additionally, when RFS was considered an event, all-cause death was regarded as a competing event. 

Propensity score (PS) matching was performed using logistic regression analysis to reduce the selection bias. Matching was performed using the following variables: age, sex, CCI, location, pathology, degree of differentiation, tumor size, and invasion depth using the nearest-neighbor algorithm at a 1:1 ratio. Matching balance was considered acceptable if the absolute value of the standardized mean difference was <0.1. Statistical significance was set at *p* value < 0.05 was considered statistically significant. Statistical analyses were performed using The R software (version 4.2.2; The R Foundation for Statistical Computing, Vienna, Austria; http://www.R-project.org/ (accessed on 3 May 2023)).

## 3. Results

### 3.1. Baseline Characteristics

Patients with superficial esophageal cancer were divided into two groups: primary surgery (*n* = 191, 75.49%) and secondary surgery (*n* = 62, 24.5%). Patients’ baseline characteristics showed significant differences in terms of invasion depth, LVI, and LNM (Table 1). The depth of invasion was greater in patients in the secondary surgery group (*p* < 0.001), and the proportion of patients with LVI was higher in the secondary surgery group than in the primary surgery group (*p* < 0.001). After PS matching, these baseline characteristics were similar, and their absolute standardized mean difference was <0.25 (Figure S1).

In the secondary surgery group, the median time from ESD to surgery was 44 days (interquartile range: 27–77). For patients who underwent ESD, the en bloc and R0 resection rates were 100% (62/62) and 74.19% (46/62), respectively. The PS-matched model revealed no significant differences between the two groups in terms of age, sex, CCI, tumor location, pathology, differentiation, tumor size, LNM, or follow-up duration.

There were three patients with M1/M2 invasion and nine with M3 invasion in the secondary surgery group. Even though they had mucosal cancer, they underwent surgery for the following reasons: lymphatic invasion (*n* = 4), incomplete resection (*n* = 5), and high risk of stricture (*n* = 2). One patient with M3 wanted to receive surgery to minimize the potential risk of cancer recurrence.

### 3.2. Survival

**Figure 2:**
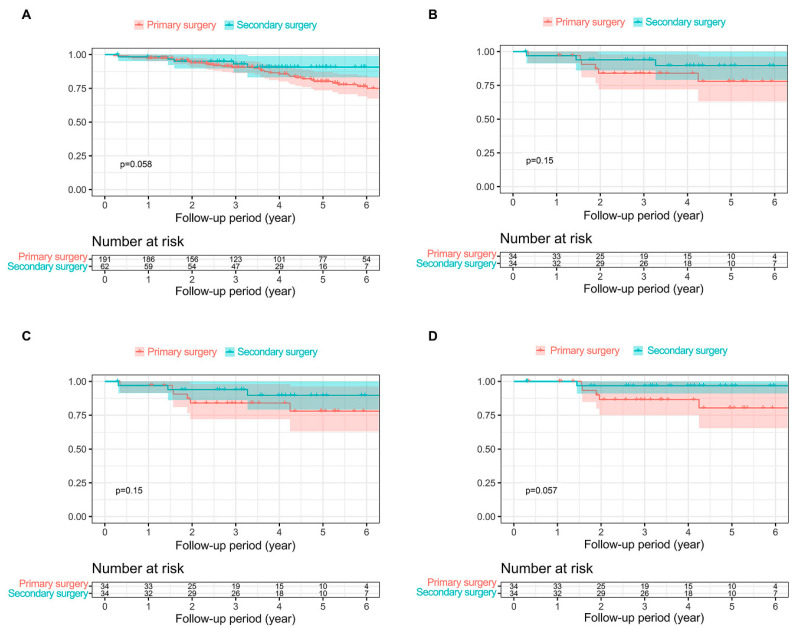
Kaplan–Meier survival curves showing overall survival and disease-specific survival. (**A**) In all patients. (**B**) In the propensity score-matched cohort. (**C**) In all patients. (**D**) In the propensity score-matched cohort.

The Kaplan–Meier survival curves for OS and DSS in all subjects (*n* = 253) and the PS-matched cohort (*n* = 68) are presented in Figure 2. The mean follow-up period for survival was 4.51 years in the total 253 patients. For the primary and secondary surgery groups, the mean follow-up periods for survival were 4.64 years and 4.13 years, respectively. There was no significant difference in OS (Figure 2A) and DSS (Figure 2C) between the primary and secondary surgery groups. 

PS-matching revealed that the mean follow-up period for survival was 4.17 years for all patients. In the primary and secondary surgery groups, it was 3.90 and 4.43 years, respectively. There was no significant difference between the PS-matched groups in terms of OS (Figure 2B) (3-year primary surgery vs. secondary surgery (84.0 (72.0–98.0)% vs. 93.9 (86.1–100)%, *p* = 0.15)) or DSS (Figure 2D) (3-year primary surgery: 86.5 (75.1–99.7)% vs. 96.8 (91.0–100)%, *p* = 0.057). 

### 3.3. Recurrence

**Figure 3:**
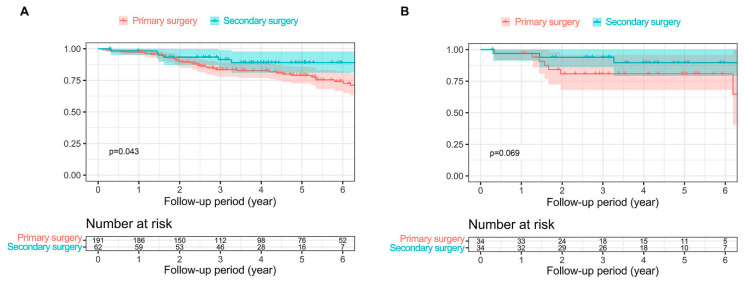
Kaplan–Meier survival curves showing recurrence-free survival. (**A**) In all patients. (**B**) In the propensity score-matched cohort.

RFS of all the participants and the matched cohort is presented in Figure 3. Although there was a statistically significant difference between the two groups for all patients (*p* = 0.043) (Figure 3A), there was none in the PS-matched cohort (*p* = 0.069) (Figure 3B). The follow-up period for recurrence for all subjects, primary, and secondary surgery groups was 4.39, 4.50, and 4.08 years, respectively. The 3-year RFS in the primary and secondary surgery group was 83.5% (78.0–89.3) and 91.4% (84.4–98.9), respectively. 

The mean follow-up period for recurrence in the PS-matched group of 68 patients was 4.15 years. The mean follow-up period for recurrence was 4.87 years and 4.43 years in the primary and secondary surgery group, respectively. There was no significant difference in RFS (the 3-year RFS for primary surgery was 80.8% (68.1–95.9) vs. 93.9% (86.1–100) for secondary surgery). 

### 3.4. Adverse Events 

Adverse events observed in the two groups are presented in Table 2. None of the patients in either group died within 60 days of treatment. There was no significant difference in the overall adverse events between the two groups. However, patients who underwent primary surgery developed complications earlier (50%) than those who underwent secondary surgery (20.6%). Patients reported various complications. The most common early complications included others (14.7%), followed by pulmonary (11.8%), cardiovascular complications (11.8%) and wound problems (11.8%). The second most common complication was leak/fistula (8.8%), followed by vocal cord palsy (2.9%). Early adverse events can also be categorized according to dindo-clavien classification (Table S1). For IIIa complications, there were pneumothorax and pleural effusion, and bleeding requiring intervention such as chest tube insertion and bleeding control. For IIIb, one patient in each group of patients had wound infection problems needing wound debridement under general anesthesia. For IV complications in the primary surgery group, there was one patient with underlying alcoholic liver cirrhosis who experienced septic shock within 30 days of surgery. This patient had multiorgan failure with sepsis and peritonitis due to gallbladder perforation, and due to the high risk of surgery, he died after 4 months of ICU care after signing a DNR (Do not Resuscitate) form. In contrast, late adverse events occurred similarly in the primary and secondary surgery groups (17.1% and 20.6%, respectively). Late complications included strictures and other complications. 

## 4. Discussion

To our knowledge, this is the first study to compare the clinical outcomes of primary and secondary surgery in patients with SESCC using a matched cohort. We used PS analysis to generate precisely matched patient cohorts. Considering data for the two groups were obtained during different time periods, surgical technology and post-operative care may differ. Therefore, we defined pathological LNM in surgical specimens as the primary outcome. LNM, OS, DSS, RFS, and adverse events in the matched cohort were comparable between the primary and secondary surgery groups.

Owing to the unique lymphatic anatomy of the esophagus, the procedure must be performed until the curative level is reached. The standard treatment is primary esophagectomy. However, as accumulating studies have shown favorable outcomes with ESD, it can be an alternative treatment for superficial esophageal cancer. For instance, the long-term outcome of esophageal ESD demonstrated excellent curability of high-grade intraepithelial neoplasms or intramucosal invasive squamous cell carcinomas (SCCs) limited to the lamina propria mucosae and a small chance of recurrence [15]. ESD provides long-term outcomes comparable with esophagectomy in patients with SESCC [16]. In addition, despite the low curative resection rate (11.8–15.2%) of ESD for T1b esophageal cancer, ESD also offers a great staging tool [17]. As the present study shows comparable outcomes between primary esophagecomy and esophagectomy after ESD, it gives more confidence for clinicians to perform ESD in patients with SESCC in the first place. 

While there is no statistically significant difference observed in terms of OS, DSS, and RFS between both groups, it is notable that secondary surgery appears to be associated with better survival outcomes. The temporal disparity in data collection for the two patient groups could have inadvertently influenced the study outcomes. There has been continuous evolution in not only surgical techniques and post-operative care practices but also pre-operative risk assessment and prevention. Advancements in pre-operative risk assessment empower clinicians to anticipate potential complications, enabling proactive preparation. Additionally, when the risk of surgical complications is deemed excessively high, clinicians have the flexibility to explore alternative treatment modalities beyond surgery. It is noteworthy that our study excluded patients who underwent concurrent chemoradiotherapy (CCRT), additional argon plasma coagulation (APC) ablation, radiotherapy (RT), and observation post-ESD. This exclusion criterion may inadvertently exclude patients with elevated surgical risk. Conversely, the unavailability of ESD before 2007 limited treatment options for esophageal cancer patients during that period. The interplay of these factors likely contributed to improved overall survival and recurrence-free survival in patients undergoing secondary surgery. 

Interestingly, in the present study, there was a higher risk of treatment-related early adverse events in patients undergoing primary surgery than in secondary surgery. The most common early complications in primary surgery were mild complications such as urethral injury, urinary retention, hypertension, subcutaneous emphysema, nausea, and post-operative delirium. Considering the time difference between the primary and secondary surgery groups, surgical technology and pre/post-operative care could have affected the differences in complication outcomes. There was one patient in the primary surgery group who had underlying liver cirrhosis and ended up experiencing multiorgan failure. A larger sample size would enhance the robustness of the statistical results. However, none of the overall patients died within 60 days of esophagectomy. The late adverse event rates were similar between the two treatment groups. The most common adverse event of ESD was stricture, which could be refractory in some cases [18,19,20]. Thus, primary esophagectomy could be optimal in patients with long and circumferential esophageal cancers.

This study had some limitations. First, a selection bias could have existed because this study was conducted retrospectively at a single tertiary center. Second, the sample size was small. Third, there may have been different exams and reports of histological evaluation of specimens from the primary surgery group than from the secondary surgery group. Endoscopically resected specimens were fixed in 10% formalin and cut into 2–3 mm-thick sections, whereas surgically resected specimens were cut into 5–6 mm-thick sections. Therefore, surgically resected specimens may undergo different pathological evaluations than endoscopically resected specimens. Finally, considering the advancements in surgery, the study period differed between the two groups. As esophageal ESD was performed after 2007, this study attempted to exclude cases of primary surgery performed before 2007. However, we had trouble analyzing the PS-matching between the two groups owing to the insufficient number of patients. In order to ensure a sufficient number, we enrolled patients of primary surgery before 2007. As two groups were obtained during different time periods, surgical technology and post-operative care may differ. In order to minimize the bias from different time periods, we defined pathological LNM in surgical specimens as the primary outcome. 

## 5. Conclusions

In conclusion, ESD before esophagectomy was comparable with primary esophagectomy in terms of LNM, OS, DSS, and recurrence. These results suggest that ESD and secondary esophagectomy can be recommended for patients with superficial esophageal cancer without compromising the outcomes, including survival, recurrence, and complications.

## Figures and Tables

**Table 1:** Baseline characteristics (primary surgery vs. secondary surgery) before and after propensity score matching.

Variables	Overall			Propensity-Score-Matched Model		
	Primary Surgery (*N* = 191)	Secondary Surgery (*N* = 62)	*p*-Value	Primary Surgery (*N* = 34)	Secondary Surgery (*N* = 34)	SMD
Age	63.0 ± 8.0	63.2 ± 7.1	0.865	62.8 ± 7.3	62.7 ± 6.3	−0.124
Sex			0.264			
Male	179 (93.7%)	61 (98.4%)		34 (100.0%)	33 (97.1%)	−0.236
Female	12 (6.3%)	1 (1.6%)		0 (0%)	1 (2.9%)	0.236
CCI			0.445			
<2	172 (90.1%)	53 (85.5%)		31 (91.2%)	30 (88.2%)	−0.083
≥2	19 (9.9%)	9 (14.5%)		3 (8.8%)	4 (11.8%)	0.083
Location of tumor			0.436			
Cervical and upper thoracic	11 (5.8%)	6 (9.7%)		1 (2.9%)	0 (0%)	−0.100
Middle thoracic, lower thoracic and EG jxn	180 (94.2%)	56 (90.3%)		33 (97.1%)	34 (100.0%)	0.100
Differentiation			0.476			
Well and Moderately	182 (95.3%)	61 (98.4%)		33 (97.1%)	34 (100.0%)	0.236
Poorly and Others	9 (4.7%)	1 (1.6%)		1 (2.9%)	0 (0.0%)	−0.236
Tumor Size	2.0 ± 1.2	2.0 ± 1.0	0.993	1.9 ± 0.9	2.0 ± 1.1	0.140
Depth 2			<0.001			
M1/2	86 (45.0%)	4 (6.5%)		4 (11.8%)	4 (11.8%)	0.000
M3	50 (26.2%)	9 (12.9%)		8 (23.5%)	7 (20.6%)	−0.087
SM	55 (28.8%)	50 (80.6%)		22 (64.7%)	23 (67.6%)	0.074
Lymphovascular invasion			<0.001			
Absent	178 (93.2%)	37 (59.7%)		28 (82.4%)	28 (82.4%)	0.000
Present	13 (6.8%)	25 (40.3%)		6 (17.6%)	6 (17.6%)	0.000
Lymph node metastasis			0.027			
Absent	171 (89.5%)	48 (77.4%)		26 (76.5%)	25 (73.5%)	-
Present	20 (10.5%)	14 (22.6%)		8 (23.5%)	9 (26.5%)	-
Follow-up duration (years)	4.6 ± 2.7	4.1 ± 2.0	0.115	3.9 ± 2.4	4.4 ± 2.4	-

Variables are expressed as mean ± standard deviation or *n* (%). CCI, Charlson comorbidity index; M1, invasion limited to the intraepithelium; M2, invasion limited to the lamina propria; M3, invasion limited to the muscularis mucosa; SM, submucosa; SMD, standardized mean difference.

**Table 2:** Adverse events (matched cohort).

	Primary Surgery	Secondary Surgery	*p* Value
60 day mortality	0	0	
Overall adverse events	20 (58.8%)	13 (38.2%)	0.145
Early Complication	17 (50.0%)	7 (20.6%)	0.021
Pulmonary	4 (11.8%)	2 (5.9%)	
CV	4 (11.8%)	1 (2.9%)	
Leak/fistula	3 (8.8%)	0 (0.0%)	
Wound dehiscence/infection	4 (11.8%)	2 (6.0%)	
Vocal cord palsy	1 (2.9%)	2 (5.9%)	
Others	5 (14.7%)	0 (0.0%)	
Late complication	6 (17.6%)	7 (20.6%)	1.000
Stricture	5 (85.3%)	7 (14.7%)	
Others	1 (2.9%)	0 (0.0%)	

Variables are expressed as *n* (%).

## Data Availability

The data that support the findings of our study are available from the corresponding authors upon reasonable request and with the permission of the respondents.

## Supplementary Materials

The following supporting information can be downloaded at: https://www.mdpi.com/article/10.3390/cancers15235542/s1, Figure S1: standardized mean difference of each variable; Table S1:early adverse events based on Dindo-Clavien classification.
