# Supplementary material for: Outcomes of Primary Esophagectomy and Esophagectomy after Endoscopic Submucosal Dissection for Superficial Esophageal Squamous Cell Carcinoma: A Propensity-Score-Matched Analysis
Source: Cancers (Basel). 2023 Nov 23;15(23):5542. doi: 10.3390/cancers15235542 (PMC10705107; doi:10.3390/cancers15235542)
Supplement: Supplementary file 1 [file cancers-15-05542-s001.zip › Table S1.docx]

**Supplementary Table S1. Early adverse events (matched cohort).**

| **Dindo-Clavian Classification** | **Primary Surgery** | **Secondary surgery** |
| --- | --- | --- |
| I | 8 (23.5%) | 3 (8.8%) |
| II | 4 (11.8%) | 2 (5.9%) |
| IIIa | 3 (8.8%) | 1 (2.9%) |
| IIIb | 1 (2.9%) | 1 (2.9%) |
| IV | 1 (2.9%) | 0 |

Variables are expressed as *n* (%).
