# Supplementary figures and images for: Outcomes of Primary Esophagectomy and Esophagectomy after Endoscopic Submucosal Dissection for Superficial Esophageal Squamous Cell Carcinoma: A Propensity-Score-Matched Analysis
Source: Cancers (Basel). 2023 Nov 23;15(23):5542. doi: 10.3390/cancers15235542 (PMC10705107; doi:10.3390/cancers15235542)

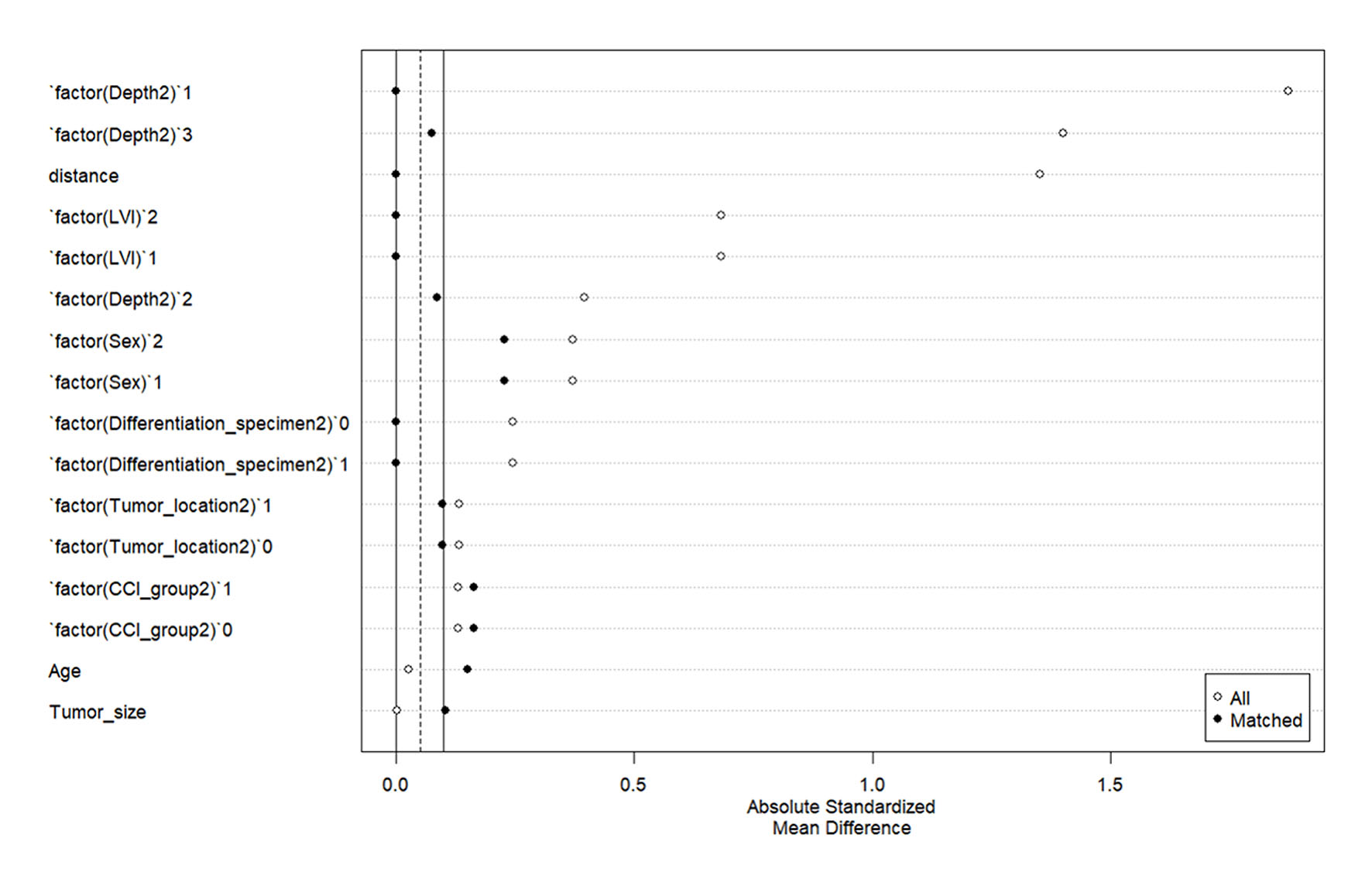

Supplement: Supplementary file 1 [file cancers-15-05542-s001.zip › Supplementary Figure S1.jpg]
